# Improving trial recruitment processes: how qualitative methodologies can be used to address the top 10 research priorities identified within the PRioRiTy study

**DOI:** 10.1186/s13063-018-2964-1

**Published:** 2018-10-25

**Authors:** Marita Hennessy, Andrew Hunter, Patricia Healy, Sandra Galvin, Catherine Houghton

**Affiliations:** 1 Qualitative Research in Trials Centre (QUESTS), School of Nursing and Midwifery, National University of Ireland Galway, Galway, Ireland; 2 Health Behaviour Change Research Group, School of Psychology, National University of Ireland Galway, Galway, Ireland; 3 School of Nursing and Midwifery, National University of Ireland Galway, Galway, Ireland; 4 Health Research Board—Trials Methodology Research Network, Galway, Ireland

**Keywords:** Documentary analysis, Focus groups, Interviews, Mixed-methods, Observation, Priorities, Qualitative, Qualitative evidence synthesis, Randomised trials, RCT, Trial methodology, Trial recruitment

## Abstract

How can we improve recruitment to trials? In their recently published paper, Healy et al. outline the top 10 prioritised questions for trial recruitment research identified by the PRioRiTy study. The challenge now is for researchers to answer these questions; but how best can these be answered? In this commentary, we illustrate how qualitative research can be utilised to generate in-depth insight into trial recruitment issues, either as a stand-alone methodology, or through a mixed-methods approach. Consideration is given to how different forms of qualitative research can be used to address these priorities and to help researchers set out an agenda to optimise its value.

## Background

In their recently published paper, Healy et al. outline the top 10 prioritised questions for trial recruitment research identified by the Prioritising Recruitment in Randomised Trials (PRioRiTy) study (Table 1) [1]. The challenge now is for researchers to answer these questions. We believe that there are significant opportunities for qualitative methodologies to contribute to better understanding of trial recruitment issues and that the true value of such methodologies has not been fully recognised, or realised, to date. By working together, all key stakeholders—including trialists, researchers, clinicians, practitioners, commissioners, managers, policy makers, and members of the public—can find answers to the various recruitment issues by embedding qualitative designs in trials, or vice versa (i.e. embedding trials within qualitative designs) [2]. To this end, this paper will present a number of examples where qualitative research has been used to improve the conduct of trials, and specifically recruitment.

**Table 1:** The “top 10” research questions prioritised in the Prioritising Recruitment in Randomised Trials (PRioRiTy) study

No.	Question
1	How can randomised trials become part of routine care and best utilise current clinical care pathways?
2	What information should trialists communicate to members of the public who are being invited to take part in a randomised trial in order to improve recruitment to the trial?
3	Does patient/public involvement in planning a randomised trial improve recruitment?
4	What are the best approaches for designing and delivering information to members of the public who are invited to take part in a randomised trial?
5	What are the barriers and enablers for clinicians/healthcare professionals in helping conduct randomised trials?
6	What are the key motivators influencing members of the public’s decisions to take part in a randomised trial?
7	What are the best approaches to ensure inclusion and participation of under-represented or vulnerable groups in randomised trials?
8	What are the best ways to predict recruitment rates to a randomised trial and what impact do such predictions have on recruitment?
9	What are the best approaches to optimise the informed consent process when recruiting participants to randomised trials?
10	What are the advantages and disadvantages to using technology during the recruitment process?

### What is the value of qualitative methods in trial recruitment research?

Qualitative research can address questions in trial recruitment that are not easily addressed by quantitative methods, by providing in-depth information on the experiences of participants and recruiters. It can also help contribute to trial design, including the development of effective recruitment strategies. Qualitative research methods have been used to address various aspects of randomised trials; these include developing and understanding the acceptability of the intervention being trialled, the trial design, process and conduct (including recruitment and retention), explaining trial outcomes, and providing contextual understanding of the target condition for the trial [3]. Increasingly, more focus is being placed on the pre-trial stage [3]. Qualitative research can potentially improve the efficiency of trials by identifying problems with recruitment. This enables the trialists to address those problems and increase or optimise recruitment [3]. Common qualitative research methods include interviews, focus groups, and observations. Other methods include analysis of trial documents, and audio recordings of trial recruitment interactions. While the integration of qualitative methods within randomised trials is recognised as important, in practice, fully embedded/integrated designs are rarely realised and methodological concerns persist [3–5]. It is imperative that researchers in primary qualitative research fully report and justify their methodological approach to ensure the rigor of their methods and maintain the credibility of qualitative research in trials. A range of potential weaknesses have been identified, including lack of clarity regarding methods, sample and data collection, limited explanation of context, poor description of data analysis, and failure to account for the impact, if any, of the qualitative researcher [6, 7] There is also a need for those undertaking qualitative evidence synthesis to address the confidence in their findings using GRADE CERQual [8]. In addition, there are reporting guidelines on the EQUATOR network (https://www.equator-network.org/) specifically aimed at qualitative research and evidence synthesis.

### How can questions be answered by qualitative methods?

We will now outline how different qualitative methodologies can be used to address the top 10 priorities. We have grouped the approaches into three categories: 1) interviews and focus groups; 2) observation, audio recording and documentary analysis; and (3) qualitative evidence synthesis. Each of the methods can also be used within mixed-methods approaches; however, we will not specifically address such approaches within this commentary. In mixed-methods research, the researcher collects and analyses both qualitative and quantitative data rigorously, integrates the two forms of data and their results, organizes these procedures into specific research designs, and frames these procedures within theory and philosophy ([9]: page 5).

#### Interviews and focus groups

The use of individual interviews or focus groups within randomised trials facilitates understanding from the viewpoint of those experiencing phenomena (in this instance, recruitment to trials), and can be conducted with patient participants, recruiters, health professionals, or others. Individual interviews provide the opportunity for in-depth discussion of individuals’ personal insights and lived experiences, particularly when the topic is potentially sensitive [10, 11]. Alternatively, the strengths of focus groups lie in group dynamics and the interactive nature of the unfolding discussions. Focus groups allow discussion in a more relaxed atmosphere to explore shared experiences and develop understanding from their interaction [12].

Donovan et al. provide a detailed example of how interviews can provide a rich understanding of the complexities and hidden challenges underlying recruitment to randomised trials from the perspectives of recruiters [13]. Similarly, Oakley et al. provide an example of the use of focus groups to support process evaluation within a trial [14]. They argue that the science of a randomised trial is enhanced by ongoing high-quality evaluation which considers the context in which the intervention is delivered, helping to explain outcomes. Analysis of focus group data provides insight into acceptability and delivery of interventions from the perspective of participants [5]; interviews can also facilitate such insights. O’Cathain et al. identify the increasing use of focus groups and individual interviews within trials, suggesting that incorporating these methods at feasibility and pilot stages of trials can enhance the leaning about trials for trialists and researchers, and contribute to the overall trial endeavour [5].

Dormandy et al. conducted interviews with general practitioners to seek their views on effective ways of recruiting and retaining practices to clinical trials [15]. This study found that interviews with general practitioners allowed identification of key strategies for communication, data collection, and payment to support retention and recruitment.

Donovan et al. synthesised findings from interviews with recruiters in a number of trials, providing improved understanding of the complexity and fragile nature of recruitment practices [13]. Interviews elicited detailed information from recruiters regarding their tendency to undermine recruitment practices, specifically randomisation for clinical and equipoise reasons [13]. Importantly, these findings were elicited in individual interviews, with responses being fed into efforts to improve recruitment practices. In an earlier study, Donovan et al. conducted in-depth interviews with men in the Prostate testing for cancer and Treatment (ProtecT) study to establish interpretation of study information by participants [2]. Subsequent changes to the content and delivery of study information within this trial, incorporating findings from these interviews amongst other data, increased recruitment rates from 40% to 70% [2]. The examples presented show that interviews and focus groups would be appropriate qualitative methods for most of the PRioRiTy questions, conscious of the group versus individual dynamic for some sensitive questions. Individual interviews would be more suitable for question nine (‘What are the best approaches to optimise the informed consent process when recruiting participants to randomised trials?’), however, where the process of informed consent would perhaps be better discussed individually.

#### Observation, audio recording, and documentary analysis

While the strengths of interviews and focus groups are to capture perspectives and experiences, there can sometimes be conflict between what people say happens and what actually happens. There are times when qualitative methods that capture interactions and events would be more suitable for answering questions about trial processes. These methods can include observations, audio recordings, and documentary analysis of trial processes. For example, Healy et al. conducted a mixed-methods process evaluation of the OptiBIRTH trial (a pan-European cluster randomised controlled trial (RCT)) [16]. An ethnographic study conducted by Maguire was embedded within this study to explore the implementation of the intervention in practice [17]. Qualitative observations and interviews uncovered the impact of the intervention on culture and rituals within the practice setting.

An example of the use of audio recording was presented by Donovan and colleagues [2]. Analysis of audiotape recordings of recruitment appointments in the ProtecT study revealed how the language used when presenting trial information could impact on recruitment [2]. This innovative qualitative approach became the cornerstone of QuinteT Recruitment Intervention (QRI) developed by Donovan and colleagues [18]. The QRI involves understanding the process of recruitment in real time and then developing an action plan to address the identified difficulties in collaboration with the RCT Chief Investigator, Trial Management Group, and Clinical Trials Unit [18].

Documentary analysis examines anything written or produced about a context and how it has evolved [19]. This can include formal and informal sources which may contain clues as to how a phenomenon has evolved [19]. This is an important method to understand what is happening and the context from which the phenomenon has grown. Documentary sources, unlike interviews, are not the result of a somewhat artificial process of interaction and, therefore, the process by which they are produced cannot be ignored [20]. Content analysis of trial documents was conducted in the Selective bladder Preservation Against Radical Excision (SPARE) feasibility study (along with analysis of interview data and audio recordings of recruitment appointments) to explore reasons for low recruitment and to attempt to improve recruitment rates [21]. Trial documents examined included the SPARE trial Patient Information Sheet and trial protocol. Findings contributed to revisions to trial processes that were acceptable to trialists and recruiters.

In recruitment research, these qualitative methods, alone or in combination (as utilized for example in the QRI), could be used to answer the “what is happening?” component of the PRioRiTy questions. For instance, using observation or audio recording, the best approaches for including under-represented or vulnerable populations (question seven) could be explored. Similarly, informed consent processes (question nine: ‘What are the best approaches to optimise the informed consent process when recruiting participants to randomised trials?’) could be examined using observations, audio recording, or documentary analysis. Researchers could also use observational techniques to explore the role of technology in recruitment processes outlined in question ten. In the context of the PRioRiTy questions, documents would be important for augmenting evidence from interviews and observations [22], to develop a better understanding about recruitment processes and outcomes.

#### Qualitative evidence synthesis

In addition to emphasising the importance of primary qualitative studies in trial research, there is now recognition of the contribution to be made by qualitative evidence synthesis (QES). QES is a valuable way of synthesising primary qualitative research to capture experiences, perceptions, and factors that impact on certain components of the trial process. QES is rigorous and provides meaningful conclusions that can inform policy and practice [23, 24].

As outlined in the PRioRiTy paper, a number of qualitative syntheses have already been conducted that address questions six and nine [25–28]. It should be noted that all of the questions identified in the PRioRiTy paper were deemed “unanswered” if there was no up-to-date systematic review (< 3 years old). Houghton et al. have also published a Cochrane Protocol exploring the factors that impact on recruitment to trials [29]. This ongoing qualitative review will be integrated with the findings of a Cochrane review [30] that aimed to identify interventions designed to improve recruitment to RCTs, which in turn will inform PRioRiTy questions two and six. QES has great potential for guiding further recommendations for most of the PRioRiTy questions.

## Conclusions: where do we go from here?

The PRioRiTy study identified and prioritised important unanswered questions on how to improve the process of recruiting people to randomised trials. Further research is now required to address those prioritised questions. We propose that qualitative research approaches have a crucial role in providing answers to the questions posed. Similar to the examples provided, qualitative research could be conducted as a stand-alone study embedded as a study within a trial (SWAT), or as part of mixed-methods research. Failure to integrate findings and variations in quality are ongoing issues [31]. Trialists and qualitative researchers need to collaborate and work together to ensure qualitative work is done appropriately, ethically, and rigorously.

As described above, there is ample opportunity for qualitative methodologies to address the top ten priorities identified for trial recruitment research. Organisations such as QUESTS, the HRB-TMRN, Trial Forge, and QuinteT can bridge links between trialists and researchers to better inform trial recruitment.

Some key considerations for moving this agenda forward are:

- Qualitative research needs to be integral, and not considered an optional add-on.
- There needs to be a common language supporting communication between trialists and researchers.
- Qualitative methodologies should be embedded at the design phase (including costings) and fully reported on completion. This includes pre-trial, pilot, and feasibility stages of the trial [3, 4].
- The potential positive impact of qualitative research in trial recruitment and other trial methodology research needs to be rigorously researched, articulated, and disseminated.

Qualitative research can help provide the necessary evidence to guide researchers on how to improve the process of how people are recruited to randomised trials; an issue which persists as a challenge to trialists.

## Abbreviations

PRioRiTy: Prioritising Recruitment in Randomised Trials
ProtecT: Prostate testing for cancer and Treatment
QES: Qualitative Evidence Synthesis
QRI: QuinteT Recruitment Intervention
QUESTS: Qualitative Research in Trials Centre
QuinteT: Qualitative Research Integrated within Trials
RCT: Randomised Controlled Trial
SPARE: Selective bladder Preservation Against Radical Excision
SWAT: Study Within A Trial
